# Orbital reconstruction in nonpolar tetravalent transition-metal oxide layers

**DOI:** 10.1038/ncomms8306

**Published:** 2015-06-24

**Authors:** Nikolay A. Bogdanov, Vamshi M. Katukuri, Judit Romhányi, Viktor Yushankhai, Vladislav Kataev, Bernd Büchner, Jeroen van den Brink, Liviu Hozoi

**Affiliations:** 1 Institute for Theoretical Solid State Physics, IFW Dresden, Helmholtzstr. 20, 01069 Dresden, Germany; 2 Joint Institute for Nuclear Research, Joliot-Curie 6, 141980 Dubna, Russia; 3 Institute for Solid State Research, IFW Dresden, Helmholtzstr. 20, 01069 Dresden, Germany; 4 Department of Physics, Technical University Dresden, 01062 Dresden, Germany

## Abstract

A promising route to tailoring the electronic properties of quantum materials and devices rests on the idea of orbital engineering in multilayered oxide heterostructures. Here we show that the interplay of interlayer charge imbalance and ligand distortions provides a knob for tuning the sequence of electronic levels even in intrinsically stacked oxides. We resolve in this regard the *d*-level structure of layered Sr_2_IrO_4_ by electron spin resonance. While canonical ligand-field theory predicts *g*_||_-factors less than 2 for positive tetragonal distortions as present in Sr_2_IrO_4_, the experiment indicates *g*_||_ is greater than 2. This implies that the iridium *d* levels are inverted with respect to their normal ordering. State-of-the-art electronic-structure calculations confirm the level switching in Sr_2_IrO_4_, whereas we find them in Ba_2_IrO_4_ to be instead normally ordered. Given the nonpolar character of the metal-oxygen layers, our findings highlight the tetravalent transition-metal 214 oxides as ideal platforms to explore *d*-orbital reconstruction in the context of oxide electronics.

## Abstract (web summary)

The iridate compounds display interesting physical properties, including quasi-two-dimensional behaviour similar to cuprates. Bogdanov *et al.* explore the *d*-level structure of Sr_2_IrO_4_ using electron spin resonance measurements and detailed calculations and find it is inverted compared to its normal ordering

## Graphical abstract

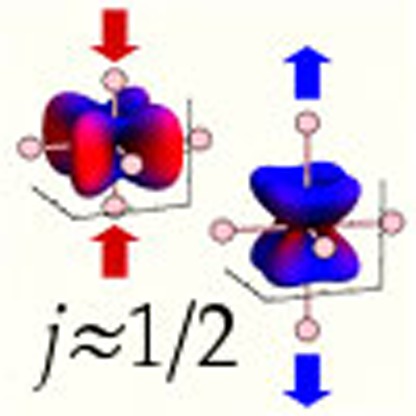

Their unique diversity of transport and magnetic properties endows transition-metal (TM) oxides with a long-term potential for applications in microelectronics and electrical engineering. Nowadays the search for new or superior properties goes beyond known bulk phases and includes oxide interfaces and stacked superlattices^1^,^2^. As compared with the bulk material, at interfaces the modification of the nearby surroundings can significantly affect the valence electronic structure, in particular, the occupation of the *d*-shell levels^1^,^2^,^3^,^4^,^5^. This is often referred to as orbital reconstruction^3^,^4^,^5^ and brings to the fore the most basic aspect in electronic-structure theory: how energy levels in quantum matter are formed and populated.

A variety of intrinsically stacked crystalline oxides is presently known. The high-temperature cuprate superconductors^6^, for example, fall in this category but also iridates of the type A_2_IrO_4_ (A=Sr^2+^, Ba^2+^) that closely resemble undoped cuprates, both structurally and magnetically^7^,^8^,^9^,^10^,^11^. Sr_2_IrO_4_ has a rather simple crystalline structure displaying stacked, quasi two-dimensional (2D) IrO_2_ and double SrO layers. We shall demonstrate that in this system the occupation of the valence *d* electronic levels differs from what is expected in textbook ligand-field theory due to electrostatics that involves both types of metal-oxygen sheets. In particular, we show that, as compared with the isostructural cuprate La_2_CuO_4_, a different distribution of ionic charges between the TM-O_2_ and A-O layers modifies the sequence of energy levels within the *t*_2g_ and *e*_g_ manifolds and consequently very fundamental physical properties such as the magnetic *g* factors, which determine the relation between the magnetic moment and quantum number of a magnetic particle. Our findings are of direct relevance to the field of stacked oxide heterostructures and provide a guideline on how low-symmetry crystal fields at *d*-metal sites can be altered and potentially engineered through the appropriate design of successive ionic layers.

To show this we first use electron spin resonance (ESR) measurements to untangle the 5*d*-shell electronic structure of crystalline Sr_2_IrO_4_, in particular, the exact order of the Ir *t*_2g_ levels. The single *s*=1/2 hole present in these *t*_2g_ orbitals carries an angular moment *l*_eff_=1 and is subject to a large spin-orbit coupling (SOC), which in first approximation results in an effective Ir^4+^ moment, or pseudospin, *j*_eff_=*l*_eff_−*s*≈1/2 (refs 7, 12, 13). We compare the experimental properties of these pseudospins with the ones we have calculated by *ab initio* quantum chemistry methods. This combined approach, explored here on a strongly spin-orbit coupled material for the first time, provides direct access to the spatial anisotropies of the *g* factors and further to the detailed microscopic superexchange interactions. The ESR measurements and theory are found to agree on a quantitative level and moreover undoubtedly show that the *d*-level ordering in Sr_2_IrO_4_ is inverted with respect to the normal ordering in the sister iridate Ba_2_IrO_4_ or the isostructural 214 cuprate superconductors. The good agreement between the ESR data and the outcome of the computational methodology we describe and employ here establishes the latter as a reliable tool for the investigation of nontrivial electronic structures and magnetic couplings.

## Results

### Pseudospins and effective Hamiltonian

**Figure 1:**
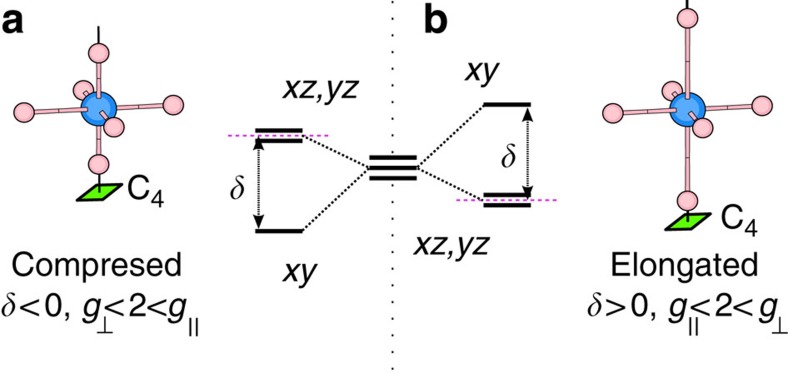
TM *t*_2g_ splittings for tetragonal distortions of the oxygen octahedron sans SOC. (**a**) *z* axis compression of the octahedron corresponds to a tetragonal splitting *δ*<0, causes an orbital dublet to be lowest in energy and the *g* factors to be ordered as 
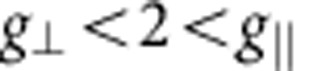
. (**b**) Elongation of the octahedron (*δ*>0) causes an orbital singlet to be lowest in energy and the *g* factors to be ordered as 
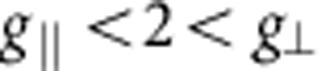
. Purple dashed lines indicate the conventional zero level used to define the sign of *δ*.

Mott-Hubbard physics in *d*-metal compounds has been traditionally associated with first-series (3*d*) TM oxides. However, recently, one more ingredient entered the TM-oxide ‘Mottness' paradigm—large SOC's in 5*d* systems. SOC in 5*d* and to some extent 4*d* anisotropic oxides modifies the very nature of the correlation hole of an electron, by admixing the different *t*_2g_ components^12^,^13^, changes the conditions for localization^7^, the criteria of Mottness and further gives rise to new types of magnetic ground states and excitations^9^,^11^. While various measurements indicate that indeed spin-orbit-coupled *j*_eff_≈1/2 states form in A_2_IrO_4_ (refs 7, 9, 14), it has been also pointed out that off-diagonal SOC's may mix into the ground state (GS) wavefunction substantial amounts of 
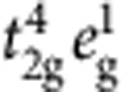
 character^15^,^16^,^17^,^18^. Such 
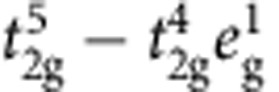
 many-body interactions were shown to produce remarkable effects in X-ray absorption and X-ray magnetic circular dichroism (XMCD): the branching ratio between the *L*_3_ and *L*_2_ Ir 2*p* absorption edges reaches values as large as 4, nearly 50% higher than the 2.75 value for a ‘pure' *j*_eff_=1/2 system^19^. In addition, low-symmetry noncubic fields produce sizeable splittings of the 5*d t*_2g_ levels, in some cases close to or even larger than ∼1/2 eV (refs 20, 21, 22), and therefore admix the *j*_eff_=1/2 and 
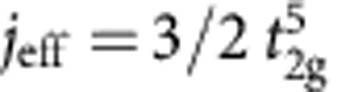
 components^13^,^15^. The structure of the spin-orbit GS depends on both the strength and sign of these splittings. Interestingly, the best fits of the X-ray absorption and XMCD data are achieved in Sr_2_IrO_4_ with a negative *t*_2g_ tetragonal splitting^19^, although the oxygen octahedra in this material display a distinct positive tetragonal distortion—the IrO_6_ octahedra are substantially elongated^23^ (a negative tetragonal splitting should occur when the IrO_6_ octahedra are compressed^13^,^24^, see Fig. 1). This is already a first indication of the level inversion that our ESR measurements and quantum chemistry calculations show to take place in Sr_2_IrO_4_.

The interactions between a pair 〈*ij*〉 of nearest-neighbour (NN) 1/2 pseudospins in the presence of an external magnetic field **h** is given by the effective Hamiltonian


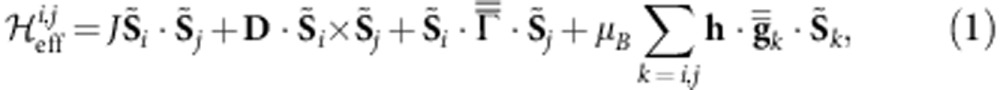


where 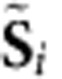, 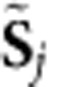 are pseudospin (*j*_eff_≈1/2) operators, *J* is the isotropic Heisenberg exchange, **D**=(0,0,*D*) defines the antisymmetric Dzyaloshinskii-Moriya (DM) coupling, 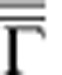 is a symmetric traceless second-rank tensor describing the symmetric anisotropy and due to the staggered rotation of the IrO_6_ octahedra the 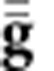 tensor splits for each of the two sites into uniform and staggered components 
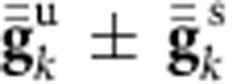
 (see for example, refs 16, 25). This effective spin Hamiltonian is of direct relevance to the interpretation of the ESR data.

### ESR measurements

**Figure 2:**
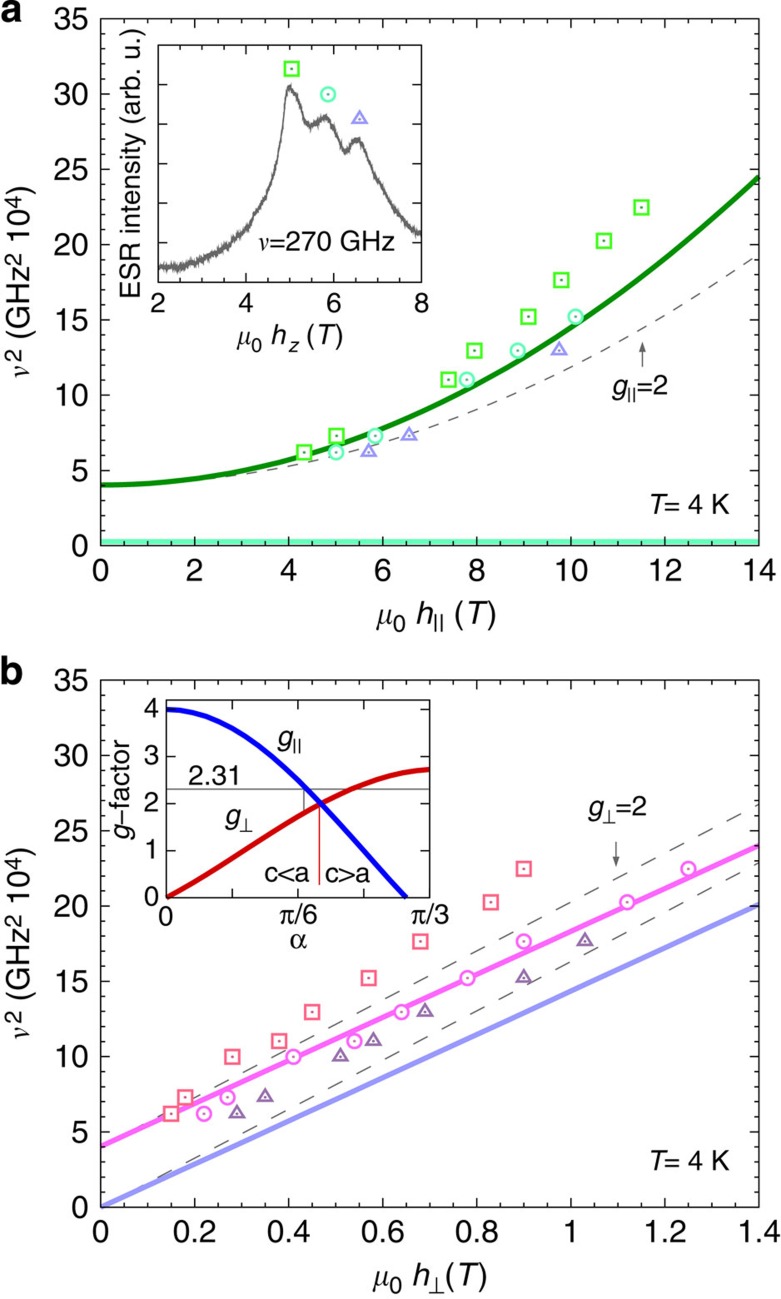
ESR data for Sr_2_IrO_4_. (**a**) Out-of-plane magnetic-field geometries; the inset shows a representative AFR spectrum. (**b**) In-plane magnetic fields; the inset demonstrates the *g*-factor anisotropy as function of the tetragonal distortion parameter *α* (see text). Symbols denote experimental data points—solid lines are theoretical curves using equations (2, 3, 4) and the quantum chemically computed *g* factors *g*_||_=2.31, *g*_⊥_=1.76 (see Table 1); dashed lines are calculated assuming isotropic *g* factors.

For a single crystal of Sr_2_IrO_4_ we observe antiferromagnetic resonance (AFR) modes in the sub-THz frequency domain^26^ as displayed in Fig. 2. There are two modes if **h**||*z*: a gapless Goldstone mode *v*_||1_=0 and a gapped excitation


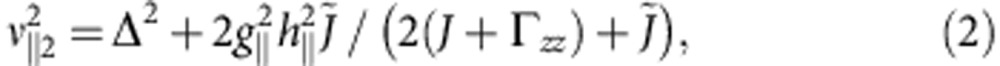


where 
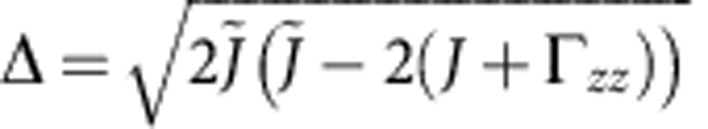
, 
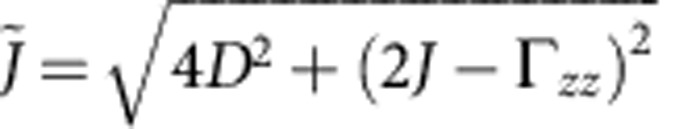
 and Γ_*zz*_ couples the 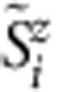 and 
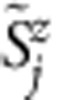
 components (along the *c* axis, perpendicular to the *ab*-plane IrO_2_ layers^23^) in the third term of equation (1) (see Methods for details). Experimental results are shown for *v*_||2_ in Fig. 2a. The data comprise at *T*≪*T*_N_=240 K a group of overlapping resonances (Fig. 2a, inset), possibly due to some distribution of internal fields in the sample. Though revealing some scatter, the 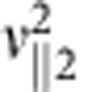 AFR data follow approximately a parabolic dependence on *h* and, most importantly, they lie substantially above the curve corresponding to the free-electron Landé factor *g*_e_=2 (dashed line in Fig. 2a). The experimental dependence 
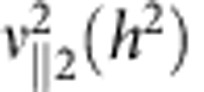
 can be reasonably well modelled with *g*_||_ values of 2.3–2.45. The solid line in Fig. 2a is obtained by using *g*_||_=2.31, as derived from quantum chemistry calculations that will be discussed later on.

Sizeable deviations to values >2 of *g*_||_ is clear indication of the presence of low-symmetry, noncubic crystal fields. In the simplest approximation, that is, restricting ourselves to the Ir^4+^ 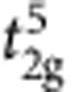 manifold, the anisotropic *g* factors in axial noncubic environment can be expressed up to the sign as^13^ *g*_||_=*g*_c_=(2+2*k*)cos^2^*α*−2sin^2^*α* and 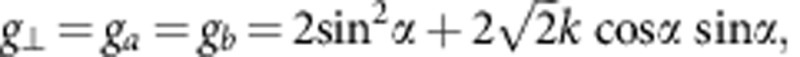 where *k* is a covalency reduction factor, *α*=(1/2) arctan 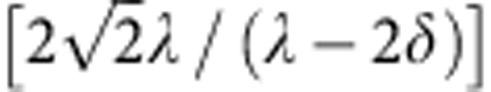 parameterizes the deviation from octahedral symmetry, *λ* is the SOC constant and δ the Ir *t*_2g_ splitting. A plot for the dependence of the diagonal *g* factors on the distortion parameter *α* is shown in the inset to Fig. 2b. For simplicity, *k*=1 is for the moment assumed but smaller values of *k* do not bring qualitative changes. In cubic symmetry *δ*=0, *α*_cub_=35.26° and the *g* matrix is isotropic with 
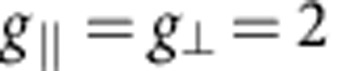
. According to standard textbooks on ligand-field theory^24^, an elongation of the out-of-plane Ir-O bond induces a positive tetragonal splitting of the Ir *t*_2g_ levels, with *δ*>0 and *α*>*α*_cub_, whereas a bond compression yields *δ*<0 and *α*<*α*_cub_ (see Fig. 1). As in Sr_2_IrO_4_ the IrO_6_ octahedra are substantially elongated in the *z* direction^23^, the *g* factors are expected to correspond to the case of positive splitting *α*>*α*_cub_, see the area to the right of the crossing point shown in the inset to Fig. 2b. It follows from the plot that *g*_||_<2, which obviously contradicts our AFR data for **h**||*z* [Fig. 2a]. The value *g*_||_=2.31 used to draw the curve connecting the open circles in Fig. 2a in fact corresponds to *α*=32.05°<*α*_cub_ (see the inset in Fig. 2b) and indicates that, despite the positive *c*-axis tetragonal distortion, a counterintuitive negative tetragonal splitting of the 5*d t*_2g_ levels is present in Sr_2_IrO_4_.

It should be noted that there is no Goldstone mode for finite in-plane magnetic fields. The canting angle depends in this case both on the strength of the DM interaction and the applied field. The two modes are


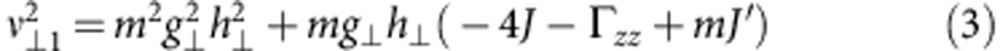


and


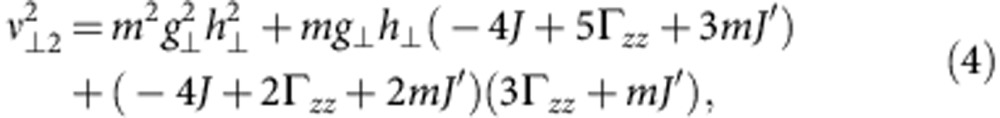


where 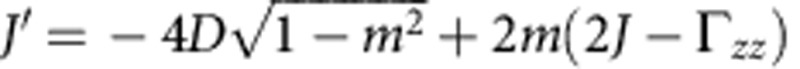 and *m* is the in-plane ferromagnetic component of the effective moments. To first order in magnetic field, *m* can be expressed as


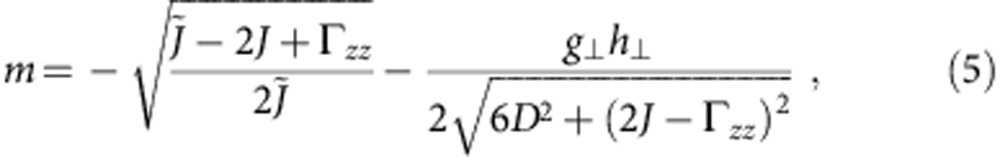


which holds for weak fields where *m* remains small. The first term corresponds to the zero-field canting, arising from the DM interaction, while the second term shows how this canting evolves with increasing *h*_⊥_. Plots based on equations (3, 4) and the quantum chemically derived interaction parameters (see below) are displayed in Fig. 2b together with ESR data.

### Quantum chemistry calculations of *g* factors

Results of *ab initio* quantum chemistry calculations for the *g* factors in Sr_2_IrO_4_ and in the structurally related material Ba_2_IrO_4_ are listed in Table 1. Our computational scheme follows the prescription of Bolvin^27^ and Vancoillie *et al.*^28^. It maps the matrix elements (MEs) of the *ab initio* Zeeman Hamiltonian 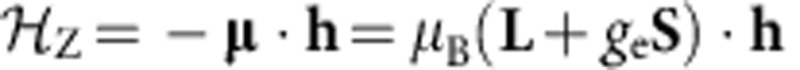 onto the MEs of the effective pseudospin Hamiltonian 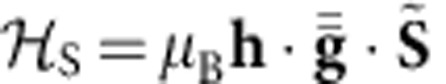, where **μ**, **L** and **S** are magnetic moment, angular-momentum and spin operators, respectively. The spin-orbit GS wave functions are computed either at the complete-active-space self-consistent-field (CASSCF) or multireference configuration-interaction (MRCI) level of theory^29^, as described in ref. 30 and using the MOLPRO quantum chemistry package^31^. All necessary angular-momentum MEs are calculated as well with MOLPRO (see Methods). In a first set of calculations, only the three *t*_2g_ orbitals at a given Ir site and five electrons were considered in the active space. The self-consistent-field optimization was carried out for the corresponding 
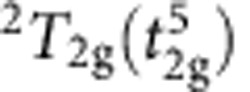
 state. We use here the more convenient notations associated to *O*_*h*_ symmetry, although the calculations were performed for the actual experimental geometry, with point-group symmetry lower than octahedral. Inclusion of SOC yields in this case a set of three Kramers doublets (KDs), see Table 1.

Subsequently we performed calculations with larger active spaces, including also the Ir *e*_g_ orbitals. One 
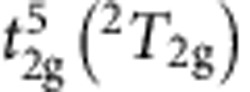
 plus four 
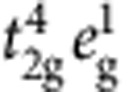
 (^2^*A*_2g_, ^2^*T*_1g_, ^2^*E*_g_ and ^2^*T*_2g_) spin doublets, two spin quartets [
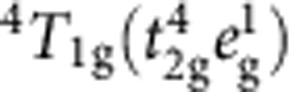
 and 
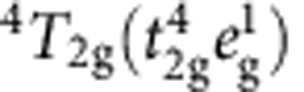
] and one spin sextet 
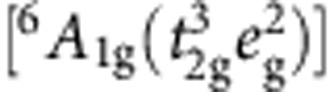
 entered here the spin-orbit treatment. The orbitals were optimized for an average of all these terms.

The effect of enlarging the active space to include 
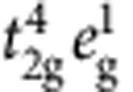
 terms in the reference wavefunction is in the range of 10%, in line with earlier semi-empirical estimates for 4*d*^5^ and 5*d*^5^ systems^15^,^16^,^17^,^18^. Most importantly, the calculations yield a negative tetragonal splitting of the Ir *t*_2g_ levels in Sr_2_IrO_4_, *δ*=−155 meV by MRCI, and positive *t*_2g_ splitting in Ba_2_IrO_4_ (see Table 1 and Methods). Similar signs, negative in Sr_2_IrO_4_ and positive in Ba_2_IrO_4_, but much larger magnitudes (≈0.7 eV) are found for the computed Ir *e*_g_ splittings (not shown in Table 1).

Taken together, the ESR and quantum chemistry results unequivocally point at an anomalous order of the split Ir 5*d* levels in Sr_2_IrO_4_, related to the important role of the extended crystalline surroundings in generating low-symmetry fields that compete with ‘local' distortions of the ligand cage. Similar effects were found by *ab initio* calculations on the 214 layered rhodate Sr_2_RhO_4_ (ref. 32) and the 227 pyrochlore iridates^22^. In contrast, in Ba_2_IrO_4_, the stretch of the apical Ir-O bonds is strong enough^33^ to overcome the longer-range electrostatics, turning the tetragonal *t*_2g_ positive again, as discussed in more detail in the following. Consequently, the structure of the 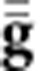 tensor in Ba_2_IrO_4_ is qualitatively different, with *g*_⊥_>2 and *g*_||_<2 (see Table 1), the ordering that one normally expects and encounters for elongated octahedra^13^.

### Exchange couplings from quantum chemistry

**Figure 3:**
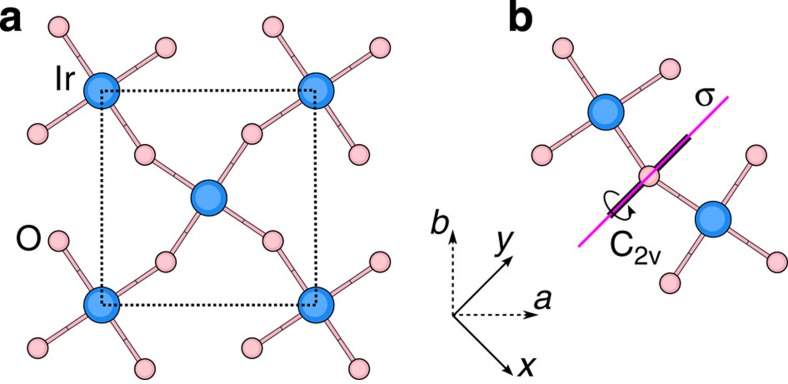
Planar IrO_2_ network in Sr_2_IrO_4_. (**a**) Coordination of the Ir site. Dashed lines show the boundaries of the crystallographic unit cell within a given IrO_2_ layer. (**b**) The point-group symmetry of the [Ir_2_O_11_] block is *C*_2v_; associated symmetry elements are indicated in the figure.

To obtain *ab initio* quantum chemistry values for the inter-site effective magnetic couplings in Sr_2_IrO_4_ (see equation (1)), we carried out additional calculations on larger clusters that incorporate two 5*d*^5^ sites. The two-octahedra cluster has *C*_2v_ symmetry (see Fig. 3), which implies a diagonal form for 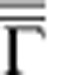 and 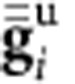 in equation (1) (see Methods). By one-to-one correspondence between the MEs of the *ab initio* Hamiltonian


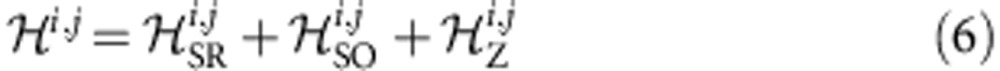


and the MEs of the effective spin Hamiltonian (1) in the basis of the lowest four spin-orbit states defining the magnetic spectrum of two NN octahedra, we can derive in addition to the *g* factors the strengths of the Heisenberg and anisotropic intersite couplings. In equation (6), 
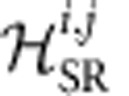
 is the scalar-relativistic Born-Oppenheimer Hamiltonian, 
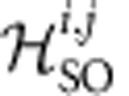
 describes spin-orbit interactions^30^ and 
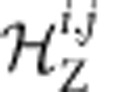
 is the two-site Zeeman Hamiltonian.

Diagonalization of the spin Hamiltonian (1) provides the expected singlet 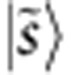 and three (split) triplet components |*t*_*x*_〉, |*t*_*y*_〉 and 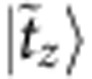. Owing to the DM interaction, 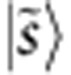 and 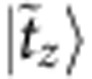 are admixtures of ‘pure' |0,0〉 and |1,0〉 spin functions. Our mapping procedure yields *J*≈48 meV, somewhat lower than *J* values of 55–60 meV derived from experiment^9^,^34^, and a ratio between the DM and Heisenberg couplings *D*/*J*=0.25, in agreement with estimates based on effective superexchange models^12^,^35^,^36^ and large enough to explain the nearly rigid rotation of magnetic moments that is observed when the IrO_6_ octahedra revolve^37^,^38^. *Ab initio* results for the NN anisotropic couplings 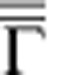, also relevant for a detailed understanding of the magnetic properties of Sr_2_IrO_4_, are shown as well in Table 2. In our convention the *x* axis is taken along the Ir-Ir link, that is, it coincides with the 〈110〉 crystallographic direction^23^, and *z*||*c*. We obtain Γ_*xx*_≈Γ_*zz*_, which then allows to recast the Heisenberg and symmetric anisotropic terms in (1) as 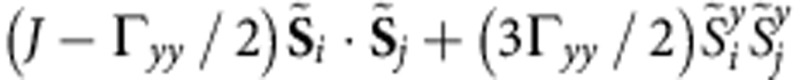, with Γ_*xx*_=Γ_*zz*_=−Γ_*yy*_/2. Equally interesting, for no rotation of the IrO_6_ octahedra and straight Ir-O-Ir bonds in Ba_2_IrO_4_, it is Γ_*yy*_ and Γ_*zz*_ which are approximately the same, providing a realization of the compass-Heisenberg model^36^,^39^ since the DM coupling is by symmetry 0 in that case.

The two-site magnetic Hamiltonian (1) features in-plane symmetric-anisotropy couplings Γ_*xx*_ and Γ_*yy*_, which were not considered in previous studies^12^,^26^. In the presence of two-sublattice order, terms containing these couplings cancel each other in the mean-field energy but they are in general relevant for pseudospin fluctuations and excitations. Using spin-wave theory and effective parameters derived from the quantum chemistry calculations, we nicely reproduce the correct GS and character of the modes, as shown in Fig. 2. To reproduce the experimental zero-field gap, in particular, we used *J*, *D* and *g*-factor values as listed in Table 2 and a somewhat larger Γ_*zz*_ parameter of 0.98 meV. To leading order, the dependence of 
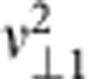
 and 
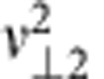
 on *h* is linear, see equations (3, 4), and the slope is proportional to *m*. At low fields (≤1 T), *m* can be actually replaced with its field independent value^26^. Using the MRCI coupling constants, the first term in equation (5) then yields a moment *m*≈0.12*μ*_B_, in good agreement with the experiment^8^,^40^.

## Discussion

**Figure 4:**
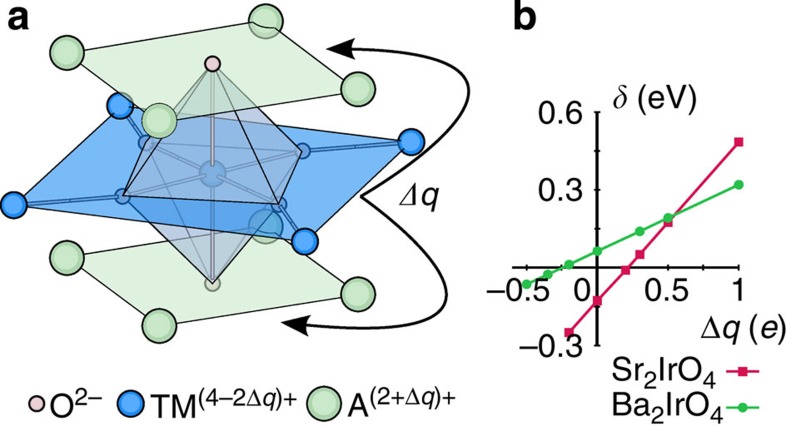
Effect of interlayer charge imbalance in A_2_IrO_4_ iridates. (**a**) The nearby surroundings of TM sites in A_2_TMO_4_-layered perovskites. In test calculations one can assign the adjacent (in-plane) TM ions the formal charge *Q*_TM_−2Δ*q*, which is compensated by assigning the NN A sites the charge *Q*_A_+Δ*q*. (**b**) Tetragonal crystal-field energy splitting between *t*_2g_ orbitals (*δ*) as a function of the charge redistribution Δ*q* for Sr_2_IrO_4_ and Ba_2_IrO_4_.

The exact *d*-level order is of fundamental importance in TM oxides, dictating for instance the symmetry of the quasiparticle states in photoemission^10^,^32^,^41^ and the nature of the magnetic ordering^42^,^43^. In 214 iridates specifically, it determines the various isotropic as well as anisotropic contributions to the magnetic exchange couplings^12^,^35^,^36^,^39^, the evolution of those magnetic interactions with strain^44^ and/or pressure^19^ and most likely the nature of the intriguing transition to a nonmagnetic phase in Sr_2_IrO_4_ under high pressure^19^. Having established that in Sr_2_IrO_4_ the *d* levels are inverted and that in the closely related Ba_2_IrO_4_ they are not raises the question what actually drives the inversion. To address this, we performed an additional set of calculations, in which we change the charges around the reference IrO_6_ octahedron. As a simple numerical experiment that preserves charge neutrality of the A_2_IrO_4_ system, we assigned the 4 NN iridium sites (in-plane, see Fig. 4) the charge *Q*_TM_−2Δ*q* and the 8 closest A-site cations (out of plane) the valence *Q*_A_+Δ*q*. In a fully ionic picture, *Q*_TM_ and *Q*_A_ are 4+and 2+, respectively. However, since in our calculations the NN TM and A sites are not modelled as just formal point charges (see Methods), the actual valence states depart from their formal values, with larger ‘deviations' for *Q*_TM_. The way we introduce Δ*q* in the computations is therefore by appropriately modifying the nuclear charge at the respective site. For variable Δ*q*, this interpolates linearly between nearby surroundings corresponding to 5*d* 214 layered perovskites (with Δ*q*=0 and TM^4+^, A^2+^ formal valence states) and their cuprate 214 equivalents (with Δ*q*=1, TM^2+^/A^3+^ formal ionic charges and ‘normal' order of the TM *t*_2g_ and *e*_g_ levels^45^).

As is illustrated in Fig. 4a, increasing Δ*q* amounts to moving positive charge from the IrO_2_ plane to the adjacent A-O layers. The calculations show that upon moving charge in such a manner, the Ir *t*_2g_ splitting *δ* increases, see Fig. 4b. In other words, this redistribution of charge counteracts the level inversion in Sr_2_IrO_4_ and further increases the already positive *δ* in Ba_2_IrO_4_. In Sr_2_IrO_4_ the cubic-like *j*_eff_=1/2 limit occurs for Δ*q*=0.22. This effect can easily be understood: placing more positive charge out of the IrO_2_ plane stabilizes the out-of-plane *t*_2g_ orbitals, corresponding to the (*yz*,*zx*) orbital doublet, and thus enhances *δ*. One can also do the opposite and drive Δ*q* negative. In this case more positive charge piles up in the IrO_2_ plane, which one expects to lower the energy of the *xy* orbital singlet, thus enhancing the level inversion in Sr_2_IrO_4_. This is indeed what happens, see Fig. 4b. What is more, driving Δ*q* negative even causes a level inversion in Ba_2_IrO_4_, when Δ*q*
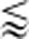
−0.25. It is interesting to note that the slope of the *δ* versus Δ*q* lines in Sr_2_IrO_4_ is much steeper than in Ba_2_IrO_4_, which is caused by the significantly smaller Ir–Ir distances in Sr_2_IrO_4_.

From these test calculations it is clear that low-symmetry crystal fields associated to neighbours beyond the first ligand coordination shell, in particular, the highly charged Ir^4+^ NNs, counteract the local tetragonal crystal field that is caused by the elongation of the IrO_6_ octahedra in both Sr_2_IrO_4_ and Ba_2_IrO_4_. In the case of Ba_2_IrO_4_ the local distortion is still strong enough to overcome these longer-range effects but in Sr_2_IrO_4_, with a slightly smaller tetragonal distortion, the longer-range electrostatics wins, causing the observed level inversion.

While the role of the high ionic charge of in-plane ions has been earlier invoked in the tetravalent Ru oxide compound Ca_2_RuO_4_ (ref. 43) and in mixed-valence manganites^46^, we here explicitly prove it by combined ESR measurements and many-body *ab initio* calculations on structurally and chemically simpler systems in which additional complications arising from further distortions^43^,^47^ or the presence of multiple TM valence states^46^ are excluded. A reversed order of the Ir *t*_2g_ levels in Sr_2_IrO_4_ has been also indirectly implied by fits of X-ray absorption^19^ and X-ray magnetic scattering^37^,^48^ spectra. As a more direct and more sensitive experimental technique to such details of the valence electronic structure and with back up from truly *ab initio* many-body calculations, ESR now provides irrefutable evidence for such physics. The numerical ‘experiment' outlined in Fig. 4 further shows that at the heart of this effect is not the intersite exchange, as assumed in ref. 19, and not the *t*_2g_-*e*_g_ orbital hybridization invoked in ref. 48, but basic interlayer electrostatics.

We have, in sum, provided an integrated picture on the *d*-level structure and magnetic anisotropies in Sr_2_IrO_4_, a prototype spin-orbit driven magnetic insulator. Both the single-site 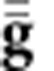 tensor and intersite effective exchange interactions are analysed in detail. To access the latter, we build on an earlier computational scheme for deriving intersite matrix elements in mixed-valence spin-orbit coupled systems^49^. While the ratio *D*/*J* of the antisymmetric Dzyaloshinskii-Moriya and isotropic Heisenberg couplings is remarkably large in Sr_2_IrO_4_ and concurs with an in-plane rotation pattern of the Ir magnetic moments that follows nearly rigidly the staggered rotation of the IrO_6_ octahedra^37^,^38^, the most prominent symmetric anisotropic terms are according to the quantum chemistry data in-plane, perpendicular to the Ir-Ir links. The structure of the 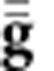 tensor, as measured by ESR and computed with first-principles electronic-structure methods, is such that 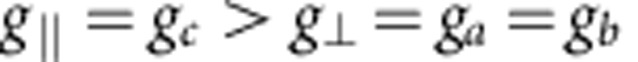 and distinctly indicates a negative tetragonal-like splitting of the Ir *t*_2g_ levels, in spite of sizable positive tetragonal distortions in Sr_2_IrO_4_. We further observe that a much stronger tetragonal distortion in Ba_2_IrO_4_ renders the tetragonal *d*-level splitting positive and *g*_||_<*g*_⊥_. The interesting situation arises that nevertheless the magnitude of the Ir *t*_2g_ splitting is largest in Sr_2_IrO_4_. The *d*-level inversion in Sr_2_IrO_4_ and the surprisingly small splitting in Ba_2_IrO_4_ have to do with the way the positive ionic charge is distributed between adjacent Ir^4+^O_2_ and A^2+^O layers, having in contrast to the 214 cuprate superconductors, for example, more positive charge in the TM-O planes. This almost compensates the ‘local' tetragonal field arising from the *z* axis elongation of the IrO_6_ octahedra in Ba_2_IrO_4_ and overcompensates it in Sr_2_IrO_4_.

The subtle interplay between local distortions of the O ligand cage and additional uniaxial fields associated with the anisotropic extended surroundings opens new perspectives on strain^44^ and pressure^19^ experiments in square-lattice iridates, for example, in connection to the spin-flop transition earlier predicted in Sr_2_IrO_4_ (refs 12, 36). It also opens up the perspective of manipulating this way the *d*-level ordering in oxide heterostructures with highly charged, trivalent and tetravalent species. Compounds with tetravalent species within the TM-O_2_ layers, in particular, given the nonpolar character of the quasi 2D sheets, provide ideal playgrounds to explore the mechanism of *d*-level ordering pointed out here since that will not be hindered by ‘interface' charge redistribution and structural reconstruction occuring in polar heterostructures from polar discontinuities^50^,^51^. A recent experimental realization of such mixed, tetravalent/divalent TM-oxide interfaces is for example the SrRuO_3_/NiO interface^52^, one system that requires in this respect closer theoretical examination.

## Methods

### Single-site magnetic properties

The *g* factors were obtained by computations on clusters which contain one central IrO_6_ octahedron, the four NN IrO_6_ octahedra and the nearby ten Sr/Ba ions. The solid-state surroundings were modelled as a large array of point charges fitted to reproduce the crystal Madelung field in the cluster region. To obtain a clear picture on crystal-field effects and spin-orbit interactions at the central Ir site, we cutoff the magnetic couplings with the adjacent Ir ions by replacing the tetravalent open-shell *d*^5^ NNs with tetravalent closed-shell Pt^4+^ 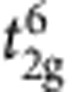 species. This is a usual procedure in quantum chemistry studies on TM systems, see for example, refs 42, 45, 53, 54, 55, 56. We used energy-consistent relativistic pseudopotentials and valence basis sets of quadruple-zeta quality supplemented with *f* polarization functions for the central Ir ion^57^ and all-electron triple-zeta basis sets for the six adjacent ligands^58^. For the TM NNs, we applied energy-consistent relativistic pseudopotentials and triple-zeta basis functions^57^ along with minimal atomic-natural-orbital basis sets^59^ for the Os coordinating those TM sites but not shared with the central octahedron. The Sr and Ba species were modelled by divalent total-ion effective potentials supplemented with a single *s* function^60^. All O 2*p* and metal *t*_2g_ electrons at the central octahedron were correlated in the MRCI calculations. The latter are performed with single and double substitutions with respect to the CASSCF reference (for technicalities, see refs 61, 62), which is referred to as MRCISD. To separate the metal 5*d* and O 2*p* valence orbitals into different groups, that is, central-octahedron and adjacent-octahedra orbitals, we used the Pipek-Mezey localization module^63^ available in MOLPRO. The computations with hypothetical (*Q*_TM_−2Δ*q*) and (*Q*_A_+Δ*q*) ionic charges at the TM and Sr/Ba sites next to the reference Ir ion were carried out as frozen-orbital multideterminant calculations (also referred to as CASCI) with three Ir *t*_2g_ and five electrons in the active space and orbitals optimized for Δ*q*=0.

The spin-orbit treatment was performed according to the procedure described in ref. 30. In a first step, the scalar relativistic Hamiltonian is used to calculate correlated wavefunctions for a finite number of low-lying states, either at the CASSCF level or at the MRCI level. In a second step, the spin-orbit part is added to the initial scalar relativistic Hamiltonian, matrix elements of the aforementioned states are evaluated for this extended Hamiltonian and the resulting matrix is finally diagonalized to yield SO wavefunctions.

The *g* factors were computed following the scheme proposed by Bolvin^27^ and Vancoillie *et al.*^28^ (for alternative formulations, see, for example, ref. 64). For the KD GS 
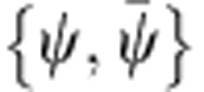
, the Abragam-Bleaney tensor^13^,^65^, **G**=*gg*^*T*^ can be written in matrix form as


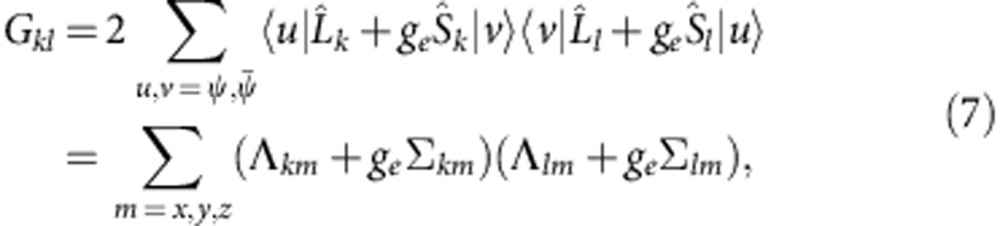


where


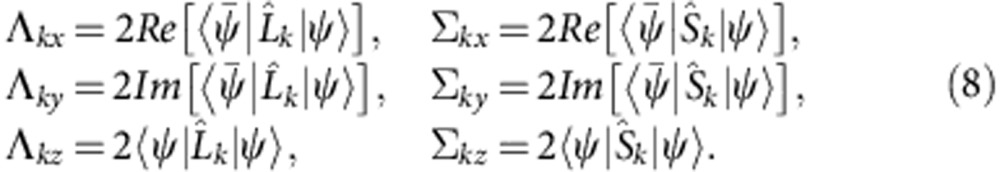


The MEs of 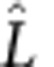 are here provided by MOLPRO while those of 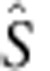 are derived using the conventional expressions for the generalized Pauli matrices :


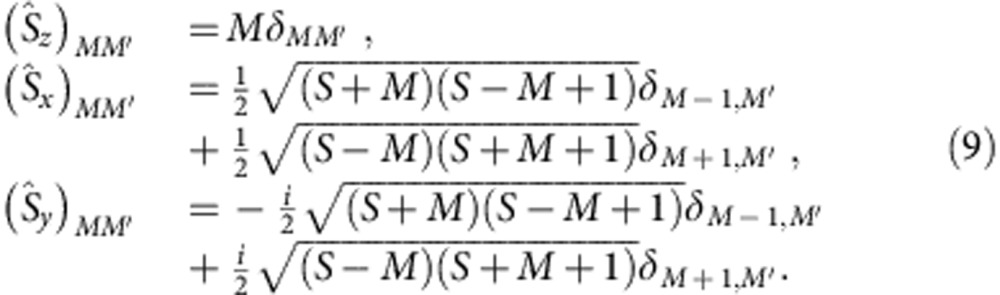


**G** is next diagonalized and the *g* factors are obtained as the possitive square roots of the three eigenvalues. The corresponding eigenvectors specify the rotation matrix to the main magnetic axes. In our case, the magnetic *z* axis is along the crystallographic *c* coordinate, while *x* and *y* are ‘degenerate' and can be any two perpendicular directions in the *ab* plane.

To cross-check the *g*-factor values computed with our subroutine, we further performed *g*-factor calculations using the module available within the ORCA quantum chemistry package^66^. We applied all-electron DKH (Douglas-Kroll-Hess) basis sets of triple-zeta quality for the TM ions^67^, triple-zeta basis functions for the ligands of the central octahedron^58^ and double-zeta basis functions for additional Os at the NN octahedra^58^. Dynamical correlation effects were accounted for by *N*-electron valence-state second-order perturbation theory (NEVPT2)^68^,^69^. CASSCF and NEVPT2 results are listed in Table 3, for both Sr_2_IrO_4_ and Ba_2_IrO_4_. It is seen that the data in Tables 1 and 3 compare very well and indicate the same overall trends.

### Superexchange interactions in Sr_2_IrO_4_

NN magnetic coupling constants were obtained for Sr_2_IrO_4_ by calculations on an embedded cluster that includes two IrO_6_ octahedra as magnetically active units. As for the calculation of single-site magnetic properties, to accurately describe the charge distribution in the immediate neighbourhood, the adjacent IrO_6_ octahedra (six) and the closest Sr^2+^ ions (16) were also incorporated in the actual cluster. We used energy-consistent relativistic pseudopotentials along with quadruple-zeta basis sets for the valence shells of the two magnetically active Ir ions^57^, all-electron quintuple-zeta basis sets for the bridging ligand^58^ and triple-zeta basis functions for the other Os associated with the two reference octahedra^58^. We further employed polarization functions at the two central Ir sites and for the bridging anion, namely 2 Ir *f* and 4 O *d* functions^57^,^58^. Additional ions defining the NN octahedra, the nearby Sr^2+^ species and the farther crystalline surroundings were modelled as in the single-site study, see above.

For two adjacent magnetic sites, the 
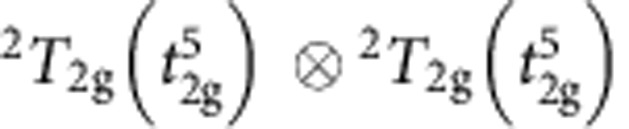
 manifold entails nine singlet and nine triplet states. The CASSCF optimization was carried out for an average of these nine singlet and nine triplet eigenfunctions of the scalar relativistic Hamiltonian 
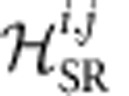
. In the subsequent MRCI treatment, only the Ir *t*_2g_ and the O 2*p* electrons at the bridging ligand site were correlated. Results in good agreement with the experimental data were recently obtained with this computational approach for related 5*d*^5^ iridates^21^,^70^.

Diagonalization of the Hamiltonian 
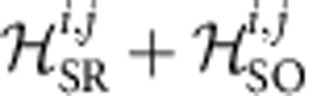
 in the basis of the lowest nine singlet and nine triplet states provides a total of 36 spin-orbit-coupled 
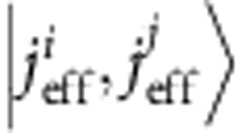
 eigenfunctions, namely, four 
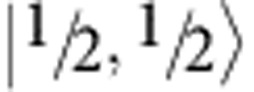
, eight 
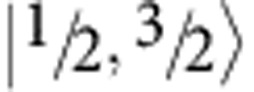
, eight 
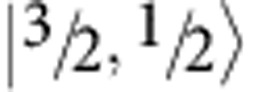
 and sixteen 
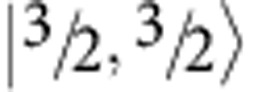
 states. In the simplest picture, the lowest four 
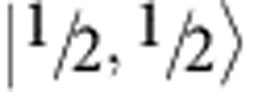
 roots imply either singlet or triplet coupling of the spin-orbit *j*_eff_=1/2 (or, more generally, pseudospin 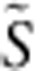
=1/2) on-site objects and are separated from higher-lying states by a gap of 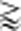
0.5 eV, much larger than the strength of the intersite exchange. It is this set of lowest four spin-orbit MRCI roots that we map onto the eigenstates of the effective two-site (pseudo)spin Hamiltonian (1). The Zeeman interaction shows up on the quantum chemistry side as 
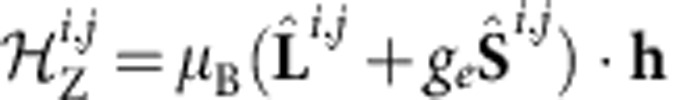
, where 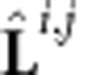 and 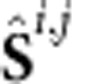 are transformed to the spin-orbit-coupled basis using the spin-orbit wavefunctions as unitary transformation matrix. MEs of the *ab initio* model Hamiltonian 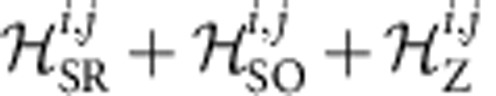 are shown in Table 4. Diagonal components show the energies of the zero-field states, while the off-diagonal MEs describe the coupling to magnetic field.

For the experimentally determined crystal structure of Sr_2_IrO_4_ (ref. 23), the two-octahedra [Ir_2_O_11_] cluster displays *C*_2*v*_ symmetry. Having the *x* axis along the 〈110〉 crystallographic direction^23^ and *z*||*c*, the effective anisotropic couplings read **D**=(0,0,*D*),


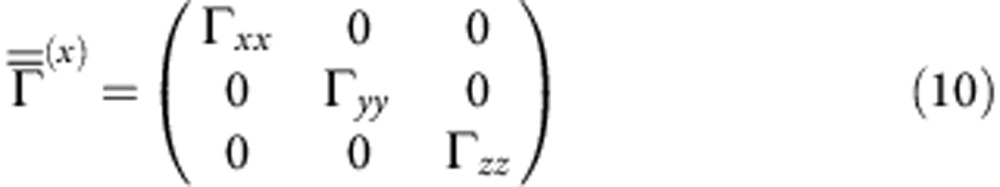


for Ir-Ir links along *x* and


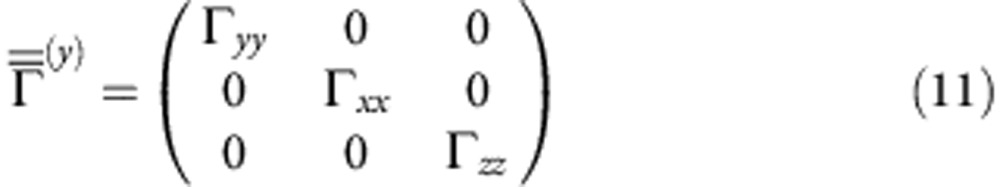


for Ir-Ir links along *y*, with Γ_*xx*_+Γ_*yy*_+Γ_*zz*_=0. The uniform and staggered components of the 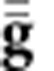 tensor take for individual Ir sites the following form :


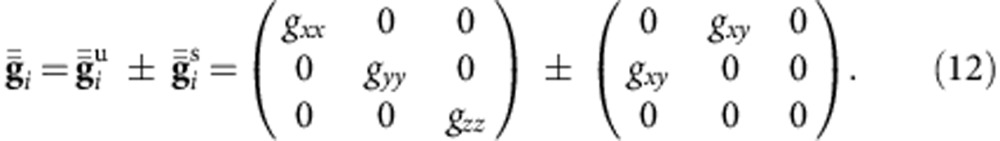


*g*_*zz*_=*g*_||_ while *g*_*xx*_ and *g*_*yy*_ are directly related to *g*_⊥_ but not restricted to be equal due to the lower symmetry of the two-octahedra cluster as compared with the IrO_6_ unit.

To solve now the actual problem, we need to transform the effective spin Hamiltonian (1) to the same form as the *ab initio* Hamiltonian shown in Table 4, that is, diagonal in zero magnetic field. The result of such a transformation is shown in Table 5. Direct correspondence between homologous MEs in the two arrays yields a set of eight independent equations that finally allow to derive hard values for all effective coupling constants that enter expression (1). While the results for the intersite exchange interactions in (1) and (10,11) are shown in Table 2, the 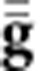-tensor data obtained from the two-octahedra calculations are *g*_*xx*_=1.64, *g*_*yy*_=1.70, *g*_*zz*_=2.31 and *g*_*xy*_=0.02. The way the additional parameters introduced in Table 5 are defined is explained in Table 6. The intermediate steps followed to arrive to the matrix form provided in Table 5 are outlined in Supplementary Tables I–III and the Supplementary Methods.

### Spin-wave calculations

For understanding all details of the ESR spectrum, we carried out a spin-wave analysis using the Hamiltonian (1) and a site-factorized variational GS wave function


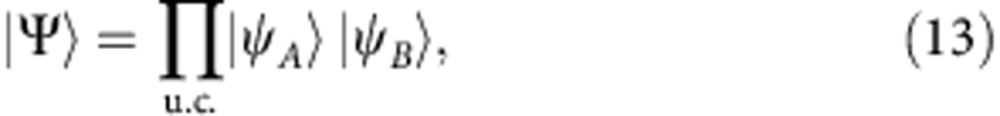


where for each sublattice index *L*∈{*A*,*B*},


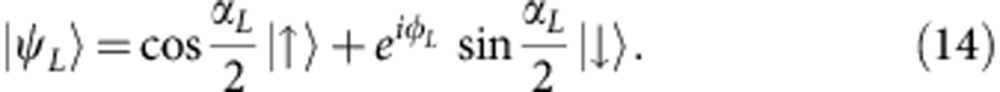


For the 2D unit cell displayed in Fig. 3a, *D*_2*d*_ symmetry is considered.

The spin components of the GS configuration depend on the *α*_*L*_ and 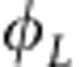 variational parameters as


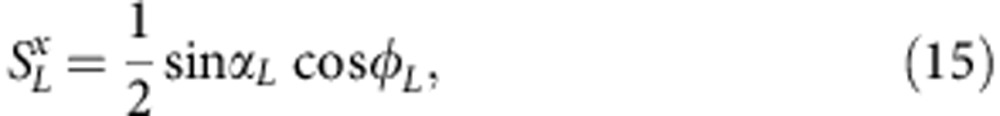



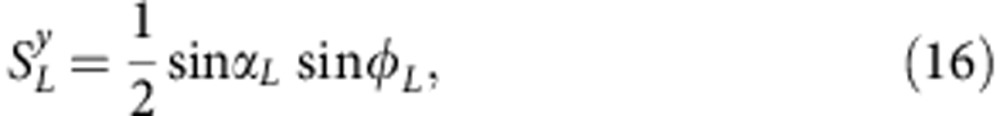



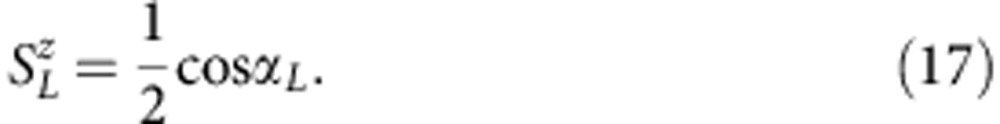


For magnetic fields parallel to the *c* axis, the GS energy only depends on the parameters *α*_*A*_=*α*_*B*_=*α* and 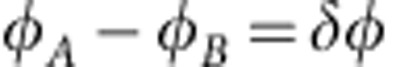. 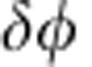 is the angle between neighbouring in-plane spins and α describes how much the spins are tilted away from the *c* axis. For *α*=*π*/2 the spins are lying within the *ab* plane, while *α*=0 corresponds to the fully polarized high-magnetic-field case. The GS energy


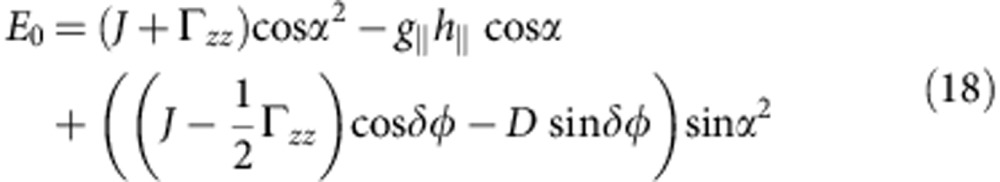


is minimized when


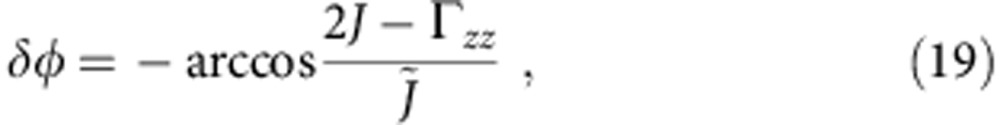



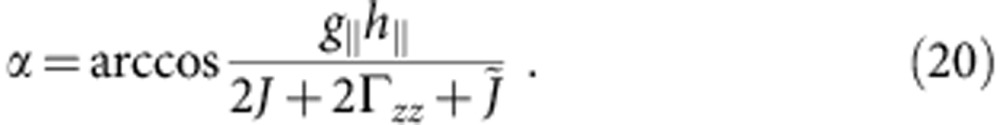


For zero field we find that *α*=0, the spins are confined to the *ab* plane (that is, to the IrO_2_ layer) and the angle 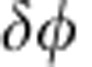 is controlled by the strength of *D*. 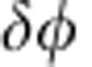 is not affected by fields along the *c* axis while α changes smoothly from *π*/2 to 0 with increasing the field strength.

The two magnons characteristic for spin-1/2 antiferromagnets are related to states orthogonal to 
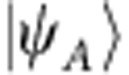
 and 
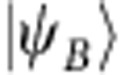
. The 4 × 4 Hamiltonian defining these magnons can be derived by using the well-known Holstein-Primakoff approach and diagonalized through a Bogoliubov transformation. For *h*||*c*, the two spin-wave modes are the gapless *v*_||1_=0 Goldstone mode, corresponding to U(1) symmetry breaking, and the gapped mode given by (2). When the magnetic field lies in the *ab* plane, the *z* component of Ir spins remains zero (with *α*=*π*/2) and the GS energy only depends on the 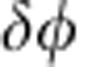 angle :


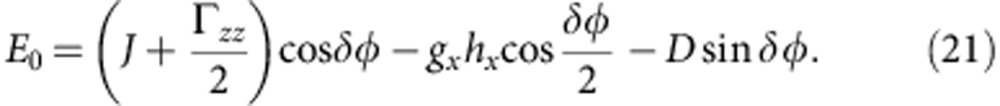


For simplicity, we select *x* for the direction of the magnetic field. The result is, however, independent of how this choice is made as there is no anisotropy within the *ab* plane.

An infinitesimally small in-plane field fixes the direction of uniform magnetization as 
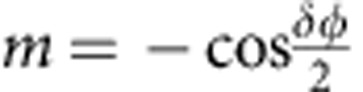
. At finite field both modes thus become gapped. To determine the uniform magnetization *m* one needs to minimize equation (21), which to leading order in magnetic field leads to equation (5). The first term of equation (5) corresponds to the zero-field moment, which arises due to the canting induced by the DM interaction. This ferromagnetic order-parameter is further enhanced in finite *h*_⊥_ field. As long as *h*_⊥_ is small equation (5) remains valid.

As discussed in the main text, in low fields, *m* can be approximated by its field independent value^26^. Using the quantum chemically derived interaction parameters (see Table 2), we then find *m*≈0.12*μ*_B_, in good agreement with recent experiments^8^,^40^. Yet the zero-field gap comes out too large as compared to experiment. A good fit can nevertheless be reached with *J*, *D* and *g* values as obtained in the MRCI treatment and by increasing Γ_*zz*_ from 0.42 to 0.98 meV.

## Additional information

**How to cite this article:** Bogdanov, N. A. *et al.* Orbital reconstruction in nonpolar tetravalent transition-metal oxide layers. *Nat. Commun.* 6:7306 doi: 10.1038/ncomms8306 (2015).

## Supplementary Material

Supplementary Information Supplementary Tables 1-3 and Supplementary Methods

## Figures and Tables

**Table 1:** *g* factors for Sr_2_IrO_4_ and Ba_2_IrO_4_.

**States considered**	**CASSCF**		**MRCI**	
	***g***_**⊥**_	***g***_**||**_	***g***_**⊥**_	***g***_**||**_
Sr_2_IrO_4_ (*δ*=−155 meV):				
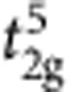 (3 KDs)	1.67	2.25	1.60	2.35
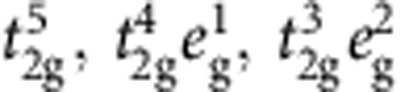 (27 KDs)	1.81	2.27	**1.76**	**2.31**

Ba_2_IrO_4_ (*δ*=65 meV):				
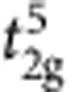 (3 KDs)	2.00	1.61	2.01	1.60
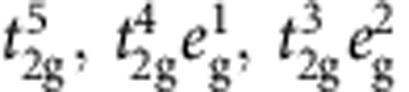 (27 KDs)	2.09	1.77	**2.10**	**1.76**

CASSCF=complete-active-space self-consistent-field; KDs=Kramers doublets; MRCI=multireference configuration-interaction. Results of many-body quantum chemistry calculations are shown. The left column displays the electron configurations entering the spin-orbit treatment. Only the high-spin sextet state is considered out of the 
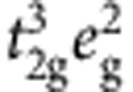
 manifold. Final results are indicated in bold face.

**Table 2:** Nearest-neighbour magnetic couplings in Sr_2_IrO_4_.

***J***	***D***	**Γ**_***xx***_	**Γ**_***yy***_	**Γ**_***zz***_
47.8	±11.9	0.42	−0.84	0.42

Results of spin-orbit MRCI calculations on two-octahedra clusters are displayed (meV). Γ_*xx*_+Γ_*yy*_+Γ_*zz*_=0 since 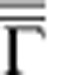 is traceless.

**Table 3:** Ir *t*_2g_ splittings and *g* factors for Sr_2_IrO_4_ and Ba_2_IrO_4_.

	***δ***_***t*****2g**_ **(meV)**	***g***_**⊥**_	***g***_**||**_
Sr_2_IrO_4_:			
CASSCF	−127	1.66	2.23
NEVPT2	−199	1.55	2.41
Ba_2_IrO_4_:			
CASSCF	30	1.93	1.74
NEVPT2	70	2.01	1.58

Results as obtained with the ORCA program^66^ are shown. Only the ^2^*T*_2g_(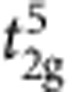) states were included in the CASSCF optimization and in the spin-orbit treatment.

**Table 4:** Matrix elements of the *ab initio* spin-orbit Hamiltonian.

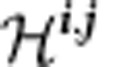	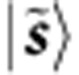	|***t***_***x***_**〉**	|***t***_***y***_**〉**	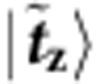
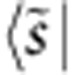	0	0.2308*iμ*_B_*h*_*y*_	−0.1768*iμ*_B_*h*_*x*_	0
〈*t*_*x*_|	−0.2308*iμ*_B_*h*_*y*_	48.3328	2.3083*iμ*_B_*h*_*z*_	−1.6854*iμ*_B_*h*_*y*_
〈*t*_*y*_|	0.1768*iμ*_B_*h*_*x*_	−2.3083*iμ*_B_*h*_*z*_	48.9626	1.6266*iμ*_B_*h*_*x*_
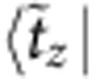	0	1.6854*iμ*_B_*h*_*y*_	−1.6266*iμ*_B_*h*_*x*_	49.0630

The latter is described by expression (6). Results of spin-orbit MRCI calculations are shown (meV). The two-site singlet and (split) triplet states are labelled 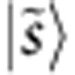 and {|*t*_*x*_〉, |*t*_*y*_〉, 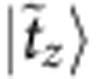}, respectively. Due to the antisymmetric exchange, 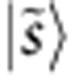 and 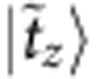 are admixtures of ‘pure' |0,0〉 and |1,0〉 spin functions.

**Table 5:** Matrix elements of the effective spin Hamiltonian.

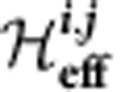	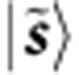	|***t***_***x***_**〉**	|***t***_***y***_**〉**	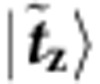
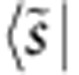	0	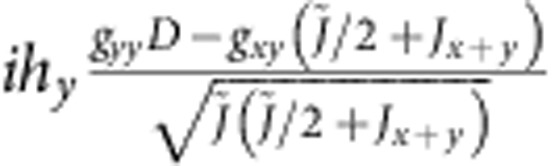	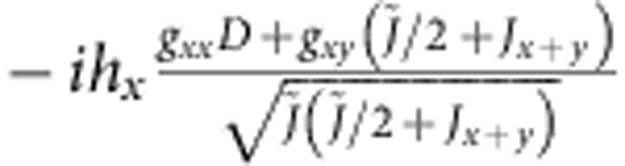	0
〈*t*_*x*_|	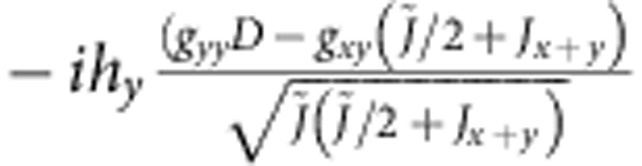	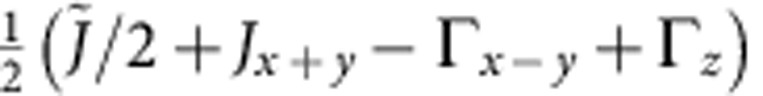	−*ig*_*zz*_*h*_*z*_	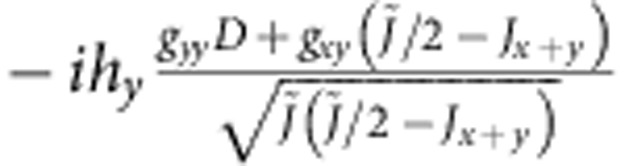
〈*t*_*y*_|	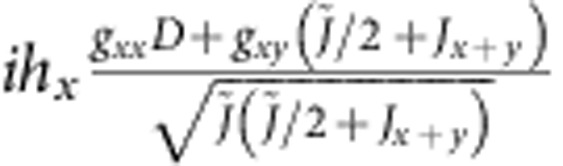	*ig*_*zz*_*h*_*z*_	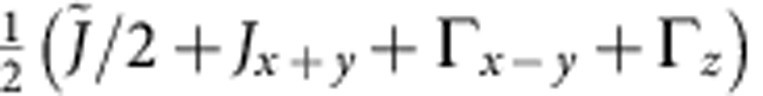	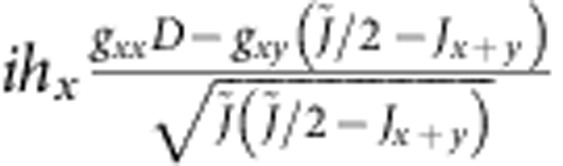
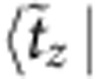	0	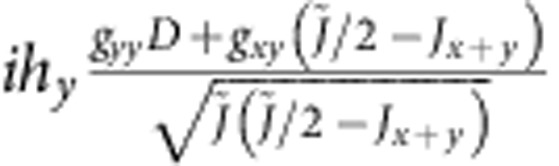	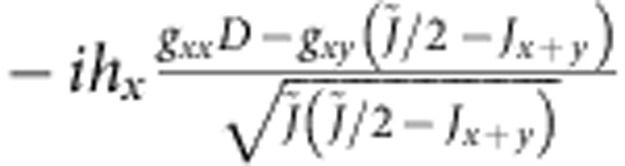	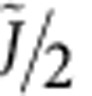

The explicit form of the latter is given by (1); for additional notations we use here, see Table 6.

**Table 6:** Notations used for anisotropic exchange coupling parameters in Sr_2_IrO_4_.

	***J*****,Γ**_***xx***_**=−Γ**_***yy***_**−Γ**_***zz***_ **(present study)**	***J***_***x***_,***J***_***y***_,***J***_***z***_ **(ref. 36)**	***J***,***δJ***_***xy***_,***δJ***_***z***_ (***x*** **bond) (refs 12,35,36)**
*J*_*x*+*y*_	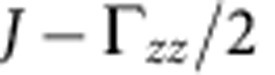	(*J*_*x*_+*J*_*y*_)/2	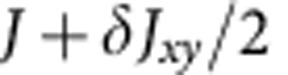
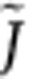	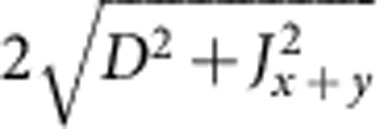	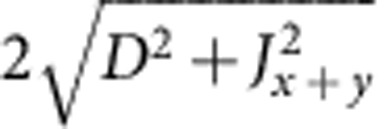	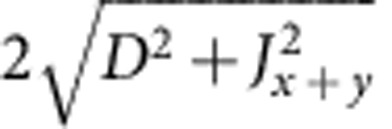
Γ_*x*−*y*_	(Γ_*xx*_−Γ_*yy*_)/2	(*J*_*x*_−*J*_*y*_)/2	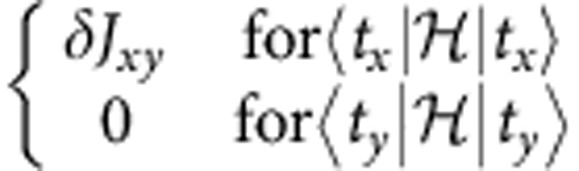
Γ_*z*_	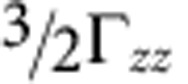	*J*_*z*_−*J*_*x*+*y*_	*δJ*_*z*_

The definitions on the left-hand side are applied in Table 5. Other conventions presently employed in the literature are also shown for comparison.
