# Supplementary material for: Orbital reconstruction in nonpolar tetravalent transition-metal oxide layers
Source: Nat Commun. 2015 Jun 24;6:7306. doi: 10.1038/ncomms8306 (PMC4491190; doi:10.1038/ncomms8306)
Supplement: Supplementary Information — Supplementary Tables 1-3 and Supplementary Methods [file ncomms8306-s1.pdf]

SUPPLEMENTARY TABLES

Supplementary Table I. **Alternative matrix form of the effective spin Hamiltonian.** The "uncoupled" bond basis $|\tilde{S}_i^z, \tilde{S}_j^z\rangle$ is here used.

$\mathcal{H}_{\rm eff}^{i,j}$	$\lvert\frac{1}{2},\frac{1}{2}\rangle$	$\lvert\frac{1}{2},-\frac{1}{2}\rangle$	$\lvert-\frac{1}{2},\frac{1}{2}\rangle$	$\lvert-\frac{1}{2},-\frac{1}{2}\rangle$
$\langle\frac{1}{2},\frac{1}{2}\rvert$	$\frac{J+\Gamma_{zz}}{4}+g_{zz}h_z$	$\frac{h_x(g_{xx}+ig_{xy})-h_y(ig_{yy}+g_{xy})}{2}$	$\frac{h_x(g_{xx}-ig_{xy})-h_y(ig_{yy}-g_{xy})}{2}$	$-\frac{2\Gamma_{yy}+\Gamma_{zz}}{4}$
$\langle\frac{1}{2},-\frac{1}{2}\rvert$	$\frac{h_x(g_{xx}-ig_{xy})-h_y(-ig_{yy}+g_{xy})}{2}$	$-\frac{J+\Gamma_{zz}}{4}$	$\frac{2J+2iD-\Gamma_{zz}}{4}$	$\frac{h_x(g_{xx}-ig_{xy})-h_y(ig_{yy}-g_{xy})}{2}$
$\langle-\frac{1}{2},\frac{1}{2}\rvert$	$\frac{h_x(g_{xx}+ig_{xy})-h_y(-ig_{yy}-g_{xy})}{2}$	$\frac{2J-2iD-\Gamma_{zz}}{4}$	$-\frac{J+\Gamma_{zz}}{4}$	$\frac{h_x(g_{xx}+ig_{xy})-h_y(g_{yy}+g_{xy})}{2}$
$\langle-\frac{1}{2},-\frac{1}{2}\rvert$	$-\frac{2\Gamma_{yy}+\Gamma_{zz}}{4}$	$\frac{h_x(g_{xx}+ig_{xy})-h_y(-ig_{yy}-g_{xy})}{2}$	$\frac{h_x(g_{xx}-ig_{xy})-h_y(-ig_{yy}+g_{xy})}{2}$	$\frac{J+\Gamma_{zz}}{4}-g_{zz}h_z$

Supplementary Table II. **Matrix elements of the effective spin Hamiltonian in the coupled basis.** The latter is described by the $|\tilde{S}_{\rm tot}, \tilde{S}_{\rm tot}^z\rangle$ functions. The zero-field couplings are shown in bold.

$\mathcal{H}_{\rm eff}^{i,j}$	$\lvert 0,0\rangle$	$\lvert 1,-1\rangle$	$\lvert 1,1\rangle$	$\lvert 1,0\rangle$
$\langle 0,0\rvert$	$-\frac{\mathbf{3}}{\mathbf{4}}\boldsymbol{J}$	$\frac{g_{xy}(h_y-ih_x)}{\sqrt{2}}$	$\frac{g_{xy}(-ih_x-h_y)}{\sqrt{2}}$	$\frac{\boldsymbol{i}}{\mathbf{2}}\boldsymbol{D}$
$\langle 1,-1\rvert$	$\frac{g_{xy}(ih_x+h_y)}{\sqrt{2}}$	$\frac{\boldsymbol{J}}{\mathbf{4}}+\frac{\boldsymbol{\Gamma_{zz}}}{\mathbf{4}}-4g_{zz}h_z$	$-\frac{\boldsymbol{\Gamma_{yy}}}{\mathbf{2}}-\frac{\boldsymbol{\Gamma_{zz}}}{\mathbf{4}}$	$\frac{g_{xx}h_x+ig_{yy}h_y}{\sqrt{2}}$
$\langle 1,1\rvert$	$\frac{g_{xy}(ih_x-h_y)}{\sqrt{2}}$	$-\frac{\boldsymbol{\Gamma_{yy}}}{\mathbf{2}}-\frac{\boldsymbol{\Gamma_{zz}}}{\mathbf{4}}$	$\frac{\boldsymbol{J}}{\mathbf{4}}+\frac{\boldsymbol{\Gamma_{zz}}}{\mathbf{4}}+4g_{zz}h_z$	$\frac{g_{xx}h_x-ig_{yy}h_y}{\sqrt{2}}$
$\langle 1,0\rvert$	$-\frac{\boldsymbol{i}}{\mathbf{2}}\boldsymbol{D}$	$\frac{g_{xx}h_x-ig_{yy}h_y}{\sqrt{2}}$	$\frac{g_{xx}h_x+ig_{yy}h_y}{\sqrt{2}}$	$\frac{\boldsymbol{J}}{\mathbf{4}}-\frac{\boldsymbol{\Gamma_{zz}}}{\mathbf{4}}$

Supplementary Table III. **Matrix form of the effective spin Hamiltonian with diagonal isotropic and symmetric anisotropic exchange.** The zero-field couplings are shown in bold.

$\mathcal{H}_{\rm eff}^{i,j}$	$\lvert s\rangle$	$\lvert t_x\rangle$	$\lvert t_y\rangle$	$\lvert t_z\rangle$
$\langle s\rvert$	$-\frac{\mathbf{3}}{\mathbf{4}}\boldsymbol{J}$	$-g_{xy}h_y$	$-g_{xy}h_x$	$\frac{\boldsymbol{i}}{\mathbf{2}}\boldsymbol{D}$
$\langle t_x\rvert$	$-g_{xy}h_y$	$\frac{\boldsymbol{J}}{\mathbf{4}}+\frac{\boldsymbol{\Gamma_{yy}}}{\mathbf{2}}+\frac{\boldsymbol{\Gamma_{zz}}}{\mathbf{2}}$	$-ig_{zz}h_z$	$-ig_{yy}h_y$
$\langle t_y\rvert$	$g_{xy}h_x$	$ig_{zz}h_z$	$\frac{\boldsymbol{J}}{\mathbf{4}}-\frac{\boldsymbol{\Gamma_{yy}}}{\mathbf{2}}$	$ig_{xx}h_x$
$\langle t_z\rvert$	$-\frac{\boldsymbol{i}}{\mathbf{2}}\boldsymbol{D}$	$ig_{yy}h_y$	$-ig_{xx}h_x$	$\frac{\boldsymbol{J}}{\mathbf{4}}-\frac{\boldsymbol{\Gamma_{zz}}}{\mathbf{2}}$

SUPPLEMENTARY METHODS

To better illustrate the nature of the $\{\tilde{s}, t_x, t_y, \tilde{t}_z\}$ states forming the basis of the effective Hamiltonian shown in Table V in the main text and the different steps followed to arrive to that particular matrix form, we also provide in this section ME's in (i) the "uncoupled" $|\tilde{S}_i^z, \tilde{S}_j^z\rangle$ basis (see Supplementary Table I), (ii) the "coupled" $|\tilde{S}_{\rm tot}, \tilde{S}_{\rm tot}^z\rangle$ basis in which the isotropic exchange is diagonal (Supplementary Table II) and (iii) the basis $\{s, t_x, t_y, t_z\}$ that also diagonalizes the symmetric anisotropic exchange (Supplementary Table III). In the latter case, $|s\rangle = |0,0\rangle$ and $|t_z\rangle = |1,0\rangle$ while $|t_x\rangle$ and $|t_y\rangle$ are antisymmetric and symmetric combinations of $|1,-1\rangle$ and $|1,1\rangle$. The unitary matrix describing the transformation to the form given in Supplementary Table III reads

$$U_\Gamma = \begin{pmatrix} 1 & 0 & 0 & 0 \\ 0 & -\frac{1}{\sqrt{2}} & -\frac{i}{\sqrt{2}} & 0 \\ 0 & \frac{1}{\sqrt{2}} & -\frac{i}{\sqrt{2}} & 0 \\ 0 & 0 & 0 & 1 \end{pmatrix}. \tag{S1}$$

Finally, $|s\rangle$ and $|t_z\rangle$ are to some degree admixed due to the antisymmetric exchange. The resulting states $|\tilde{s}\rangle$ and $|\tilde{t}_z\rangle$ imply a "rescaling" of the Zeeman couplings. The Hamiltonian thus takes the form shown in Table V in the main text. The corresponding transformation matrix reads

$$U_D = \begin{pmatrix} -\frac{iD}{\sqrt{\tilde{J}\left(\tilde{J}/2-J_{x+y}\right)}} & 0 & 0 & \frac{iD}{\sqrt{\tilde{J}\left(\tilde{J}/2+J_{x+y}\right)}} \\ 0 & 1 & 0 & 0 \\ 0 & 0 & 1 & 0 \\ \sqrt{\frac{\tilde{J}/2-J_{x+y}}{\tilde{J}}} & 0 & 0 & \sqrt{\frac{\tilde{J}/2+J_{x+y}}{\tilde{J}}} \end{pmatrix}. \tag{S2}$$
